# The Law and Infectious Disease

**Published:** 1903-10

**Authors:** 


*EDITOR:*

WILLIAM WARREN POTTER, M. D.

All communications, whether of a literary or business nature, books for review and exchanges should be addressed to the editor: 284 FRANKLIN STREET, BUFFALO, N. Y.

VOL. XLIII.—LIX. OCTOBER, 1903. No. 3.

### The Law and Infectious Disease.

PROPHYLAXIS in medicine is surely growing more in favor with both laity and profession, and even by the courts, who look upon the dissemination of disease, knowingly and willingly by the afflicted as a misdemeanor, punishable by fine and imprisonment.

During the past few months several announcements have been made in the press of such action by the courts relative to the propagation of venereal diseases, as the two following items will show:

The Munich courts have condemned to five months' imprisonment a post-office employee who had established intimate intercourse with a chambermaid of good character ("ein unbescholtenes Zimmermadchen"), although he knew that he was suffering from an uncured venereal disease.

It its column on medical jurisprudence the *Journal de Paris* narrates the following:

It is interesting to give, *in extenso,* the text of a judgment rendered by the tribunal of the Seine, on January 29, 1903. It relates to the transmission of syphilis to a young girl under age of consent, and civil damage as a consequence.

It is established by correspondence, and notably by a letter that is to be attached to this judgment, the aforesaid letter being written by X, the 21st of July, 1901, besides other grave charges, that are authentic and agreed on, that (1) in the earlier months of the year 1901, X was attacked by syphilis; (2) that he had at the same time, from the month of February to the month of July, 1901, sexual relations with a young girl that was under age; (3) that this girl, in due time, was attacked by syphilis in the month of June, 1901.

Agreed, under these circumstances, it is sufficiently well established that X has given the young girl his contagious malady knowingly, etc.

The verdict goes on to give the good and valid reasons of the court for granting the wronged girl damages, and the conclusion of the decision says, for the reasons enumerated, X is condemned to pay the plaintiff, 12,000 francs ($2,400) damages, and all the expenses of the trial.

It is interesting to note that both of these cases occurred in centers noted for their lack of moral cleanliness, and it is all the more gratifying to know that in these very centers the wronged have some recourse to justice, even if not to public opinion.

Civil suits for damages occupy the greater attention of our over busy courts, and in the majority of cases as the result of slight accident, functional nervous disturbances only ensue. No permanent lesion follows, the case is stubbornly fought and the award acts as a most powerful tonic, insuring an early convalescence.

How different is it with the infection of a venereal disease. At best it is a question of months, even years before perfect health is regained, and that at a great financial and physical loss. If liability is conceded in the one case, why not in the other where manifold more injury is inflicted? The Paris and Munich courts of tribunal need imitators throughout the entire civilised world.

